# Hemoglobin transfusion trigger in an internal medicine department – A "real world" six year experience

**DOI:** 10.1371/journal.pone.0193873

**Published:** 2018-03-07

**Authors:** Naomi Rahimi-Levene, Tomer Ziv-Baran, Victoria Peer, Ahuva Golik, Abraham Kornberg, Ronit Zeidenstein, Maya Koren-Michowitz

**Affiliations:** 1 Blood Bank, Assaf Harofeh Medical Center, Zerifin, Israel; 2 Hematology Institute, Assaf Harofeh Medical Center, Zerifin, Israel; 3 Sackler Faculty of Medicine, Tel Aviv University, Tel Aviv, Israel; 4 Department of Epidemiology and Preventive Medicine, School of Public Health, Sackler Faculty of Medicine, Tel Aviv University, Tel Aviv, Israel; 5 Internal Medicine Department A, Assaf Harofeh Medical Center, Zerifin, Israel; Providence VA Medical Center, UNITED STATES

## Abstract

**Background:**

Transfusion guidelines advocate restrictive rather than liberal use of red blood cells (RBC) and are based mostly on randomized trials in intensive care and surgical departments.

We aimed to study RBC transfusion practice in the medical patients' population.

**Methods:**

The data in this study were collected from patients over the age of 18 years admitted to an Internal Medicine department between 2009 and 2014 who received at least one unit of packed red blood cells (RBC). In addition, data on demographics, patients' diagnoses, laboratory tests and number of transfused RBC units were extracted from the electronic health records.

**Results:**

One thousand three hundred and twenty eight patients were included, having mean age of 75 ± 14 years. The median hemoglobin (Hb) trigger for RBC transfusion was 8.0 g/dl (IQR 7.3–8.7g/dl), and most patients received either one (43.4%) or two (33.4%) RBC units. There was no significant difference in Hb trigger between males and females (Hb 8.0 g/dl and 7.9 g/dl, respectively, p = 0.098), and a weak correlation with age (r = 0.108 p = 0.001). Patients with cardiovascular and lung diseases had a statistically significant higher Hb trigger compared to patients without those diagnoses, however the median difference between them was 0.5 g/dl or less.

**Conclusions:**

These "real world" data we collected show a Hb trigger compliant with the upper limit of published guidelines and influenced by medical patients' common diagnoses. Prospective trials addressing patients hospitalized in internal medicine departments could further contribute to transfusion decision algorithms.

## Introduction

Patient Blood Management (PBM) is an evidence based multidisciplinary approach to optimising care of patients in need of transfusion [1]. It aims to improve clinical outcomes by avoiding unnecessary exposure to blood components. With increasing evidence of transfusion-related adverse outcomes, the hemoglobin (Hb) at which packed red blood cells (RBC) transfusion is beneficial, the Hb trigger, was first addressed in the innovative TRICC trial [2]. Hb transfusion triggers were further defined by Carson et al [3] as liberal compared to restrictive with Hb thresholds of 10 g/dl and 8 g/dl, respectively. The literature supports similar or better outcomes with the restrictive strategy compared to the liberal one [4]. Accordingly, the American Association of Blood Banks (AABB) published clinical practice guidelines on RBC transfusion in 2012 which suggest adhering to a restrictive policy [5]. Internal Medicine departments treat patients with a wide range of diagnoses, both acute and chronic, many of whom require RBC transfusion during hospitalization. Interestingly, only a few studies include data on transfusion in Internal Medicine departments, and most prospective clinical trials were conducted in the intensive care units (ICU) or on patients undergoing surgery.

This work examined the RBC transfusion policy in an Internal Medicine department during a period of six years between 2009 and 2014.

## Materials and methods

Assaf Harofeh Medical Center (AHMC) is an 850 bed teaching hospital in central Israel, treating an urban and rural population of approximately one million people. The Internal Medicine Division includes 7 Internal Medicine Departments, each with a capacity to admit 45 patients.

This is a retrospective, observational, cross sectional study from a single Internal Medicine department cohort. The study was approved by the Assaf Harofeh Medical Center institutional review board (IRB) and the requirement for informed consent was waived, due to the retrospective nature of the study.

### Study population

**Fig 1:**
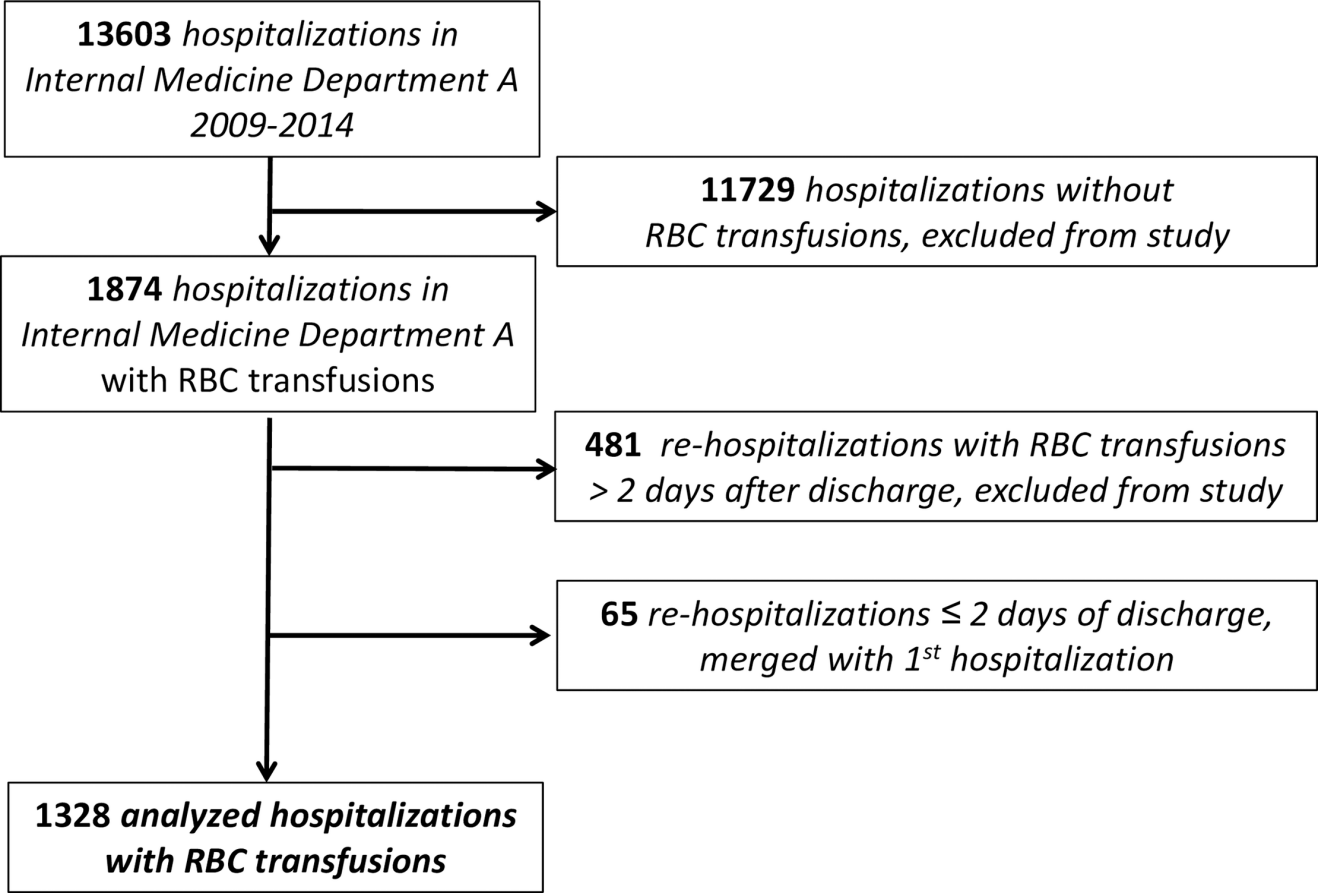
Study design flowchart.

All patients aged 18 years and above that were hospitalized in Internal Medicine "A" department and received a transfusion of at least one unit of RBC between January 2009 and December 2014 were included in the study. Patients and final study group composition are shown in Fig 1. There were 13603 patients hospitalized in Internal Medicine department "A" during the study period, 11729 of them did not receive RBC transfusions and therefore were excluded from the study. There were 1879 hospitalizations of patients with administration of RBC transfusions, 481 of them were rehospitalized within more than 2 days and were excluded from the study. Thus only the first hospitalization during the study period was analysed. Sixty five patients were discharged and rehospitalized within 2 days, and these rehospitalisations were considered to be the same event (Fig 1).

The blood bank database was used to identify the study population. In addition, clinical data including demographics, patients' diagnoses, laboratory tests and the number of RBC transfused were collected from the hospital electronic health records (EHR).

Patients' diagnoses were grouped into major categories in order to minimize inaccuracies, and were based on the International Classification of Diseases 9^th^ Revision Clinical Modification, (ICD-9-CM). All relevant diagnoses were grouped by a senior physician specializing in Internal Medicine and Hematology (N.R.L.).

Hb trigger was derived from the complete blood count (CBC) closest to the RBC transfusion and within 24 hours prior to the transfusion. Additional pre-transfusion laboratory results were taken from the tests closest to the transfusion.

### Statistical analysis

Categorical variables were described using frequency and percentage. Continuous variables were evaluated for the normal distribution using histogram and Q-Q plots. Normally distributed continuous variables were described by their mean and standard deviation, while non-normally distributed continuous variables were described by their median and interquartile range (IQR). Continuous variables were compared between groups using an independent sample t-test or a Mann-Whitney test. Correlation between continuous variables was evaluated using the Spearman's rank correlation coefficient. All statistical analysis was 2-tailed, and p values were adjusted for multiple comparisons using FDR methods, with p<0.05 being considered statistically significant. Chi-Squared Automatic Interaction Detection (CHAID)[6] is a decision tree technique for partitioning data into homogeneous groups, creating a tree where each leaf (node) is the predicted target category. Categories that are not significantly different are merged into a single node. Possible cross tabulations for each categorical predictor are performed until the best outcome is achieved and no further splitting can be performed. The relationships between the split variables and the associated related factor within the tree are visualized. CHAID modelling was used to identify characteristics of the study population significantly associated with the transfusion trigger. The statistical analysis was performed using SPSS (IBM Corp. Released 2015. IBM SPSS Statistics for Windows, Version 23.0. Armonk, NY: IBM Corp).

## Results

There were 1328 patients who received RBC transfusions in the final analysis, with a mean age of 75±14 years, and 639 (48%) males. The median hospitalization length was seven days (IQR 3–16 days). RBC transfusions were given at a median of 2 days after hospital admission. For patients receiving more than one RBC transfusion, the last transfusion was at a median of 4 days after hospital admission.

The normal Hb range in our hospital is 13.5–17.5g/dl. The median Hb trigger in the entire cohort was 8.0 g/dl (IQR 7.2–8.6 g/dl). There was no significant difference in Hb trigger between males and females (Hb 8.0 g/dl and 7.9 g/dl, respectively, p = 0.098). Further, only a weak correlation between age and Hb trigger was found (r = 0.108 p = 0.001).

Hb triggers according to patients' morbidities are presented in Table 1. Patients with respiratory diseases (including asthma, chronic obstructive pulmonary disease and lung infections) and cardiac disorders (including chronic ischemic heart disease and congestive heart failure) had a statistically significant higher transfusion trigger compared to patients without those diagnoses. However the maximal difference between the median Hb triggers of these patients compared to patients with other diagnoses was low (0.5 g/dl).

**Table 1:** Hb trigger according to patients' diagnoses.

Diagnosis category	Patients N (%)	Median Hb trigger g/dl (IQR)		p value^a^
		With diagnosis	Without diagnosis	
**Infections**	395 (29.7)	8.0 (7.3–8.7)	8.0 (7.2–8.7)	0.808
**Pneumonia, Influenza and Bronchiectasis**	234 (17.6)	8.2 (7.6–8.7)	8.0 (7.2–8.6)	**0.025**
**Respiratory Diseases Asthma and COPD**	287 (21.6)	8.4 (7.7–8.9)	7.9 (7.2–8.6)	**0.008**
**Sepsis**	145 (10.9)	7.9 (7.35–8.6)	8.0 (7.3–8.7)	0.917
**Ischemic Heart Disease**	222 (16.7)	8.3 (7.6–8.9)	8.0 (7.2–8.6)	**0.008**
**Congestive Heart Failure**	140 (10.5)	8.5 (7.8–8.9)	8.0 (7.2–8.6)	**0.008**
**Cerebrovascular Disease**	105 (7.9)	8.2 (7.5–8.2)	8.0 (7.3–8.6)	0.096
**Arterial Disease**	46 (3.5)	8.3 (7.3–9.2)	8.0 (7.3–8.6)	0.917
**Solid Neoplasms**	173 (13.0)	8.1(7.3–8.6)	8.0 (7.3–8.7)	0.917
**Hematologic Malignancies and Diseases**	125 (9.4)	7.9 (7.2–8.6)	8.0 (7.3–8.7)	0.380
**Endocarditis**	41 (3.1)	8.4 (7.8–8.9)	8.0 (7.3–8.6)	0.060
**Rheumatic Heart Disease**	15 (1.1)	7.8 (7.4–8.9)	8.0 (7.3–8.7)	0.917
**Coagulation Defects**	64 (4.8)	8.1 (7.2–8.8)	8.0 (7.3–8.6)	0.900
**Hypercoagulation and Thrombosis**	40 (3.0)	8.05 (7.4–8.8)	8.0 (7.3–8.7)	0.882
**Pulmonary Embolism**	13 (1.0)	8.3 (7.6–9.2)	8.0 (7.3–8.7)	0.523
**All Patients**	1328 (100)	8.0 (7.3–8.7)		

^a^After FDR adjustment

Pre-transfusion blood tests other than Hb level are given in Table 2. Although there were statistically significant correlations between Hb trigger and other blood tests (Table 2), they were weak (r<0.15) and therefore were defined as non-clinically significant.

**Table 2:** Pre-transfusion laboratory tests and correlation with the hemoglobin transfusion trigger.

Laboratory test		Median (IQR)	R	p
**Blood count**	White blood cells count 10^3^/μl	8.9 (6.2–13)	0.164	<0.01
	Platelets 10^3^/μl	223 (158–302)	-0.018	0.507
**Blood chemistry**	Urea mg/dl	67 (43–110)	0.060	0.061
	Creatinine mg/dl	1.29 (0.87–2.11)	0.067	0.036
	Alkaline phosphatase U/L	78 (62–107)	0.077	0.109
	Aspartate aminotransferase, AST U/L	21 (15–33)	0.078	0.105
	Alanine aminotransferase, ALT U/L	15 (10–24)	0.121	0.012
	Bilirubin total mg/dl	0.4 (0.3–0.7)	0.078	0.100
	Bilirubin direct mg/dl	0.7 (0.4–1.2)	0.054	0.675
	Albumin g/L	33 (28–37)	0.053	0.294
	Lactic Dehydrogenase, LDH U/L	401 (317–526)	0.045	0.358
**Ancillary tests**	C reactive protein, CRP mg/L^a^	80 (19–164)	-0.060	0.503
	Troponin ng/ml^b^	0.04 (0.02–0.09)	0.019	0.765

^a^Normal CRP values 0.3–5 mg/L

^b^Normal Troponin values <0.03ng/ml

**Fig 2:**
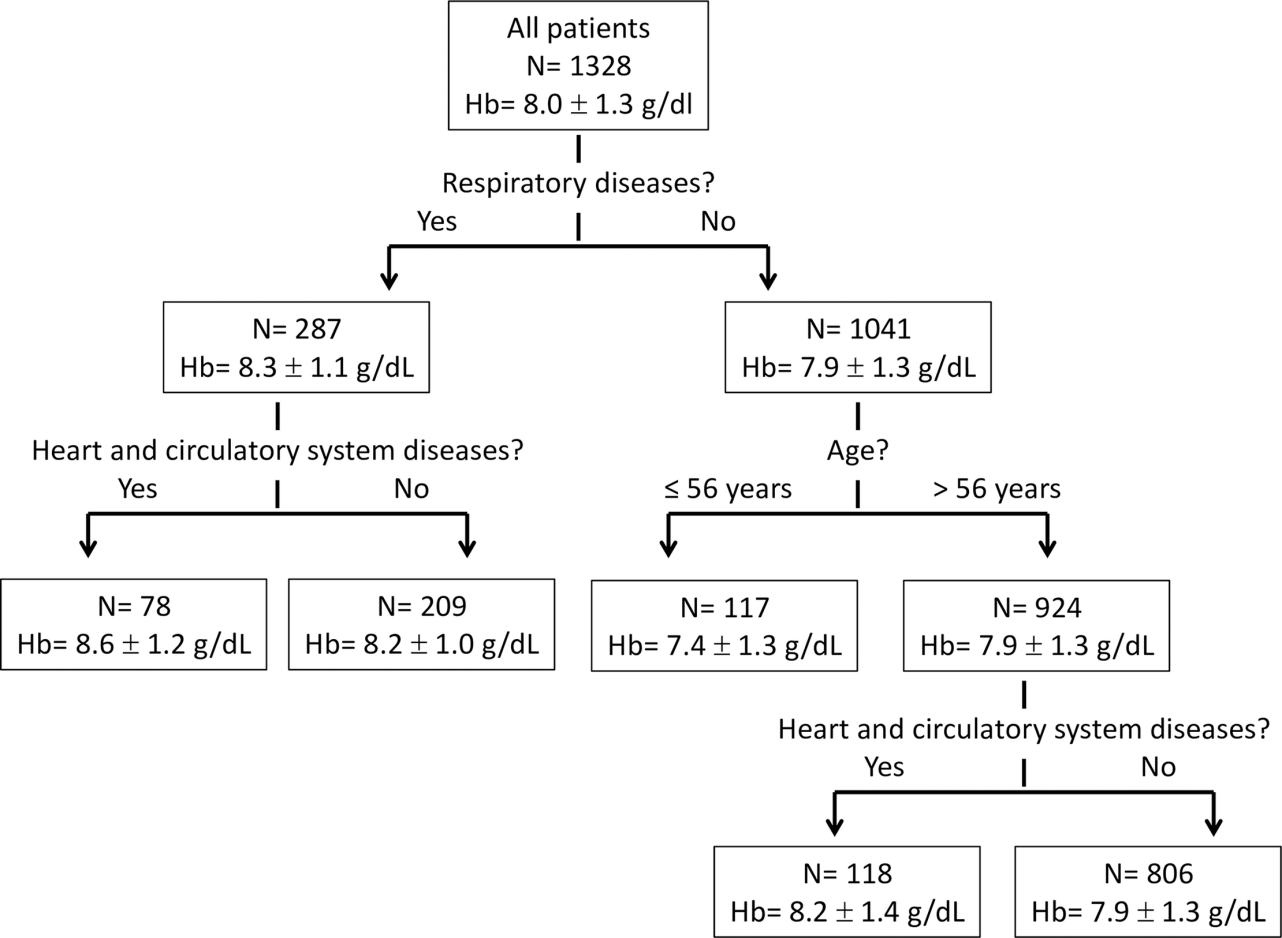
CHAID model of characteristics influencing transfusion.

According to the CHAID model, respiratory and heart diseases, in addition to age, were the major contributing parameters in the process leading to RBC transfusion (Fig 2). The lowest mean transfusion trigger of 7.4g/dl was in patients with no respiratory diseases, 56 years old or younger. The highest mean transfusion trigger of 8.6g/dl was in patients with respiratory diseases as well as heart or circulatory system diseases.

The number of RBC units transfused per patient is shown in Table 3. One and two units of RBCs were given in 43.4% and 33.4% of patients, respectively. Thus, approximately three quarters of the patients transfused received 1–2 units of RBCs during hospitalisation.

**Table 3:** Number of RBC units per patient.

RBC Units (N)	Patients N (%)	Cumulative Percent
1	577 (43.4)	43.4
2	443 (33.4)	76.8
3	173 (13)	89.8
4	66 (5)	94.8
5	24 (1.8)	96.6
6	16 (1.2)	97.8
7–42	29 (2.2)	100
Total	1328 (100)	

Twenty nine (2.2%) of the patients received more than 7 units of RBC. Ten of these patients had acute gastrointestinal bleeding, 13 were oncology patients (6 hemato-oncology and 7 oncology patients). Of the 29 patients, 18 deceased during the hospitalization in the study period. The median length of stay in this group of patients was 43 days (IQR 17–62).

## Discussion

This study describes Hb transfusion trigger of all patients hospitalized in an Internal Medicine department over a 6 years period and shows that restrictive transfusion practice was undertaken in most patients. The median Hb trigger was 8.0 g/dl, and most patients received 1–2 RBC units. These “real world” data we collected were in accordance with the AABB guidelines even before their publication [5]. In our study, patients with respiratory and cardiac diseases had a statistically significant higher Hb transfusion trigger compared to patients without those diagnoses. We also found using the CHAID model that respiratory diseases, heart diseases and age, were associated with RBC transfusion (Fig 2). The lowest mean transfusion trigger of 7.4 g/dl was in patients with no respiratory diseases, 56 years old or younger, and highest mean transfusion trigger of 8.6 g/dl in patients with respiratory diseases as well as heart or circulatory system diseases, regardless of age. Carson and Rutgers are currently performing a study comparing liberal and restrictive transfusion strategies for patients who have had an acute myocardial infarction and are anemic ((Myocardial ischemia and transfusion study (MINT), NCT02981407, NIH clinical trials site), it will be interesting to see the effect of ischemic heart disease on transfusion strategy.

The AABB recommendations for the *Choosing Wisely* campaign of the American Board of Internal Medicine are to use a restrictive threshold of 7–8 g/dl for the vast majority of hospitalised, stable patients [7]. However, most available published data report on patients in critical care or a postoperative setting, while there is less evidence regarding hospitalised medical patients. More specifically, the lowest suggested Hb threshold of 7 g/dl was derived from studies conducted in the ICU context [5]and may not apply to all patients in Internal Medicine departments.

Support of restrictive therapy was first shown in the TRICC trial, a randomized prospective landmark study, which coined the terms restrictive versus liberal transfusion therapies. Patients hospitalised in the ICU were randomly assigned to RBC transfusion at Hb thresholds of either 7 g/dl (restrictive strategy) or 10 g/dl (liberal strategy) [2].Their conclusions were that a restrictive transfusion strategy was at least as effective, possibly superior to a liberal one in critically ill patients with normovolemia. Carson et al randomly assigned hip fracture patients with cardiovascular disease or risk factors to RBC transfusion at Hb threshold of either 8 g/dl or 10 g/dl. Their results show that there was no difference in the rates of death or inability to walk independently on a 60-day follow-up between the two transfusion strategies [8]. Villanueva et al randomly assigned patients with upper gastrointestinal bleeding to a Hb threshold of 7.0 g/dl versus 9.0 g/dl showing improved outcomes with the lower threshold at 45 days including mortality and re-bleeding[9]. Holst et al. randomized ICU patients with septic shock to receive blood transfusion at a Hb cut-off of either 7 or 9 g/dl, showing no difference in 90 days mortality rates between the two groups [10]. Data on Hb transfusion trigger in medical patients was included in several case cohort studies, and summarized in a recent meta-analysis [11]. Restrictive transfusion strategy with a Hb cut-off of 7.0 g/dl significantly reduced clinical endpoints including cardiac events, re-bleeding, bacterial infections, and total mortality compared to higher Hb triggers.

The Hb trigger of 8.0 g/dl found in our study is at the higher range of transfusion threshold recommended by the AABB, and is similar to observational cohort reports from the same study period. Surial et al reported on Hb thresholds in all transfused patients in a single center during 15 months period. The Hb trigger in the Internal Medicine departments was 7.3 g/dl and was significantly lower compared to the surgical departments and the ICU [12]. Frank et al compared transfusion strategies in in-hospital services during a 44 months period. The mean Hb trigger in Internal Medicine departments (defined as the lowest Hb level during hospitalization) was found to be 7–8 g/dl [13]. Roubinian et al reported a significant decline of inpatient pre-transfusion Hb over a 5 years period, from 8.1 g/dl to 7.6 g/dl, in both surgical and medical patients [14]. This decrease occurred after the implementation of transfusion initiatives including educational sessions and hospital transfusion guidelines.

In accordance with the guideline on a lower Hb trigger, the AABB *Choosing Wisely* campaign states: "*Don’t transfuse more units of blood than absolutely necessary"*, with the guiding principle "less is more"[7]. They suggest transfusing one unit of RBC in nonbleeding hospitalised patients, reassessing the patient both clinically and with repeat Hb and only then to decide whether to transfuse further units of RBC. Published data on the number of transfused RBC units is limited. Avdic et al found that transfusing one unit of RBC to autologous hematopoietic stem cell transplant patients was non-inferior to 2 units, with a trend to non-inferiority in allogeneic transplant hematopoietic stem cell transplant patients [15]. Shehata et al reported on the number of transfused RBC units in hospitalised patients over a four and a half year period [16]. Hb triggers and patients morbidities were not reported in that study, and the mean number of transfused RBC units per patient in Internal Medicine departments was 3.2. We found that 43% of patients were transfused with one unit of RBC, and 33% received 2 units. Only few patients received 3 RBC units or more and they were mainly bleeding, hemato-oncology and oncology patients. This is within the scope of the AABB recommendations and seems encouraging since it was achieved outside of a clinical trial. Twenty nine (2.2%) of the patients received more than 7 units of RBC, and the median length of stay in this group of patients was 43 days (IQR 17–62). This may account for some of the excessive blood transfusion in this small group of patients.

Limitations of the current study are the retrospective collection of data. Grouping of diagnoses was done according to the diagnoses in the patients EHR, and we were not able to reassess the hemodynamic stability of each individual patient or study the influence of RBC transfusion of the patients' symptoms.

## Conclusion

These "real world" data we collected show Hb trigger compliant with the upper limit of published guidelines. Restrictive use of RBC transfusion was implemented in the Internal Medicine department even before the publication of clinical practice guidelines, with most patients receiving one to two units of RBC during hospitalization. Patients' symptoms and comorbidities could be main factors in a RBC transfusion approach in the older age medical population. In addition, older patients with respiratory and cardiac diseases may require transfusions at a slightly higher Hb level than other patients. Prospective trials assessing patients' symptoms prior and post RBC transfusion, diseases and clinical outcomes could further assist in appropriate decision making.
